# Bats, Pathogens, and Species Richness

**DOI:** 10.3390/pathogens10020098

**Published:** 2021-01-21

**Authors:** Frédéric Dutheil, Maëlys Clinchamps, Jean-Baptiste Bouillon-Minois

**Affiliations:** 1 CNRS, LaPSCo, Physiological and Psychosocial Stress, Université Clermont Auvergne, 63000 Clermont-Ferrand, France; fdutheil@chu-clermontferrand.fr; 2 Preventive and Occupational Medicine, University Hospital of Clermont-Ferrand, 63000 Clermont-Ferrand, France; mclinchamps@chu-clermontferrand.fr; 3 CHU Clermont-Ferrand, 63000 Clermont-Ferrand, France; 4 CNRS, LaPSCo, Physiological and Psychosocial Stress, Emergency Medicine, University Hospital of Clermont-Ferrand, 63000 Clermont-Ferrand, France

**Keywords:** bat, zoonoses, pandemic

## Abstract

Bats carry many viruses, but this is not sufficient to threaten humans. Viruses must mutate to generate the ability to transfer to humans. A key factor is the diversity of species. With 1400 species of bats (20% of all species of mammals), the diversity of bats species is highly favorable to the emergence of new viruses. Moreover, several species of bats live within the same location, and share advanced social behavior, favoring the transmission of viruses. Because they fly, bats are also hosts for a wide range of viruses from many environments. They also eat everything (including what humans eat), they share humans’ environment and become closer to domestic species, which can serve as relays between bats and humans. Bats also have a long-life expectancy (up to 40 years for some bats), which is particularly effective for transmission to humans. However, a recent publication came out challenging what we think about bats. Proportionally, bats may not carry a higher number of zoonotic pathogens, normalized by species richness, compared to other mammalian and avian species. Viral zoonotic risk is homogenous among taxonomic orders of mammalian and avian reservoir hosts, without evidence that bats carry more viruses that infect humans.

Bats have an unfavorable reputation. This may be due to looking like devils [1], having a dark color and being nocturnal. They like the dark. Perhaps also because bats are linked to the original source of transmission of Ebola (caused by *Ebolavirus*) [2], Nipah (*Henipavirus*) [3] and many coronaviruses (*Severe Acute Respiratory Syndrome, Middle East Respiratory Syndrome*) [4]. Bats have recently been suspected of representing the origin of the COVID-19 pandemic in 2020 [5]. Coincidently, the French translation of bat is “chauves-souris”, i.e., the anagram of “souches-à-virus” or “virus strains”. However, is this bad reputation justified? Bats have characteristics that can actively support the emergence of new viruses. However, a recent publication challenged some of our understandings of the negative impact of bats, challenging us to revisit the biological and social evolution of bats [6].

First, bats carry many viruses [7]. They carry almost eight times more families of viruses than rabbits and hares. This is a very strong peculiarity that confirms that bats have a unique immune system among mammals [7]. Usually, when someone is infected by a virus, the body fights the viral replication by generating an immune response, i.e., inflammation. Inflammation is the natural defensive reaction against intruders [8]. The more powerful this reaction is, the more effective the impact. However, when the inflammation is too strong, it may damage the cells of the body that are normally engaged for virus protection. Exacerbated inflammation can worsen the disease [9]. Remarkably with bats, their inflammatory reaction to a viral infection is weaker than other mammals [10]. Despite the intrusion of viruses, bats are less likely to become sick and therefore less likely to transmit the virus to others. This particular ability to be “tolerant” to viruses has been acquired by bats over the course of their evolution [7].

This immune responses among bats could also be related to the fact that bats fly [11]. Compared to birds [12], flying takes a lot of effort for mammals, it is less efficient and requires a great deal of energy, i.e., a lot of oxygen [13]. Oxygen can be dangerous for cells, by generating oxidative stress through the production of reactive oxygen species (ROS) [14]. Unstable molecules can attack cells and DNA [9]. The DNA can therefore be damaged and spread to sites where it is not supposed to be. Misplaced DNA that is out of its intended place, looks a lot like a virus. The frequent presence of these pieces of DNA that look alike to viruses, may ensure that bats have developed a less aggressive immune system [7]. As a result, when real viruses infect them, their response is more moderate, which would allow them to resist many diseases [15].

However, carrying a lot of viruses does not necessarily correlate with the potential of these viruses to be dangerous to other species. To endanger humans, viruses need to obtain the ability to be transmitted to humans. They must mutate and therefore produce new strains/lineages and—with time—new species. A key factor is the diversity of species. There are more than 1400 species of bats [16]; one fifth of the mammal species [17]. When a bat carries a virus, it replicates in its body. During this replication process, some of the produced virus copies are not completely identical [18,19]. The virus has thus mutated. Sometimes these mutations allow it to adapt to other types of bats. The virus is subsequently no longer the same. The new virus can be transmitted to other species and then mutate again to adapt to others. Increased diversity in bat species ensures more possibilities for virus mutation. Therefore, with bats accounting for 20% of all species of mammals, the diversity of bat species is highly favorable to the emergence of new viruses.

Moreover, in addition to the number of species, bats are sympatric, i.e., they engage in numerous forms of contact within and among different species of bat [20]. Many different species of bat can share the same ecological niche [21]. For example, in Brazil some species, such as colonies of colossus bats, live in very large numbers of up to 3000 individuals per square meter, and millions in a single site [22]. On top of this, bats are innately tactile. They have well advanced social behavior [23], such as social grooming including licking each other [24], within and between different species of bats; favoring the transmission of viruses.

Bats have other peculiarities. They live much longer than other light-weight mammals, so they have more time to catch viruses [10]. For example, bats of the species *Myotis brandtii* only weigh 4 to 8 g as adults, and have a life expectancy of 41 years [25]. In other words, a small animal weighing only 4 to 8 g in adulthood can live longer than a cow or a horse [26], and competes with a dolphin in terms of life expectancy [27].

Bats have also an exceptional capacity for hosting a wide range of viruses from multiple environments. As they have a wide variety of diets, bats access diverse environments for food. This diversity exposes them to more viruses [21]. Their flying capabilities ensure bats can further increase the size of the areas where they can be exposed to viruses. To reiterate, multiple characteristics of bats favor a priori the emergence of viruses in bats.

Of significance, is how these characteristics of bat biology and habitation can endanger virus distribution in human beings. Similar to virus replication with bats, transmission occurs among bat species. This exacerbates the potential for genome mutations and may result in the new viruses sometimes adapting to other species. Some of these viruses may adapt to humans directly or to another species first—an intermediate host such as a dromedary or a pangolin. As such, the virus mutates within the intermediate host and then adapts to humans. Intermediate hosting was also exemplified in MERS-CoV [28], another coronavirus which killed hundreds of people in 2012. It was also observed in EBOLA (a hemorrhagic fever) [29], rabies [30] and COVID-19. Bats are also particularly effective for transmission to humans because they live for a long time, they eat everything (including what humans eat), and they fly, which is ideal for moving closer to human installations and also closer to domestic species, which can serve as a relay between bats and humans.

Despite all previous arguments, bats do not really transmit more than all other animals. Even if bats are considered as reservoirs of several zoonoses, Mollentze et al. demonstrated that virus species within a bat reservoir (*Coronaviridae*, *Paramyxoviridae*, and *Filoviridae*) are no more likely to be zoonotic than those transmitted by other hosts [6]. The proportion of human-infecting viruses is rather constant across reservoir taxonomic orders, but the number of zoonoses carried by a group of animals (for example bats, hares and rabbits) is correlated with the diversity of species within each group. Hares and rabbits have fewer species, and subsequently less zoonoses. Conversely, rodents carry more zoonotic viruses, but there are also a greater number of species in this group. Therefore, the number of viruses that can infect humans increases concomitantly to the overall number of viruses within each reservoir group, itself explained by the number of species within each animal group. Bats may not carry a higher number of zoonotic pathogens when normalized to account for species richness, compared to other mammalian and avian species [6].

On average, bat viruses do not appear to infect humans more than viruses from other animals. Of the 54 viruses they carry, just under half can be transmitted to humans. Rodents species are similar: of the 105 viruses they harbor, just under half of them can cause zoonoses. Here too, a proportionality relationship emerges. Even, primates (56 viruses) or artiodactyla (including pigs (112 viruses) or cows, for example), carry more zoonoses [6]. There is no evidence that intrinsic or ecological differences among animal groups increases the number of viruses hosted or the likelihood that such virus become zoonotic. Indeed, viral zoonotic risk is homogenous among taxonomic orders of mammalian and avian reservoir hosts, without evidence that viruses infecting humans are more often carried by bats [6].

In conclusion, bats do carry many viruses, some of which are potentially dangerous to humans. Yet, proportionally, they carry the same number of zoonotic viruses as other animals. Bats are not responsible for carrying excessive numbers of viruses. We must keep in mind that the culprit is the human being. With the encroachment of our urban, agricultural, and industrial habitat modifications into wild spaces, humanity has moved closer to the habitats where bats live. In other words, humans have consequently moved closer to viruses [31]. Destructive reactions to bats in response to the COVID-19 pandemic could be misguided.

## References

[1] Johnson N., Hirose J.M. (2018). The impact of paralytic bovine rabies transmitted by vampire bats in Latin America and the Caribbean. Rev. Sci. Tech. l’OIE.

[2] Leendertz S.A.J., Gogarten J.F., Düx A., Calvignac-Spencer S., Leendertz F.H. (2016). Assessing the Evidence Supporting Fruit Bats as the Primary Reservoirs for Ebola Viruses. EcoHealth.

[3] Gaudino M., Aurine N., Dumont C., Fouret J., Ferren M., Mathieu C., Reynard O., Volchkov V.E., Legras-Lachuer C., Georges-Courbot M.-C. (2020). High Pathogenicity of Nipah Virus from Pteropus lylei Fruit Bats, Cambodia. Emerg. Infect. Dis..

[4] Valitutto M.T., Aung O., Tun K.Y.N., Vodzak M.E., Zimmerman D., Yu J.H., Win Y.T., Maw M.T., Thein W.Z., Win H.H. (2020). Detection of novel coronaviruses in bats in Myanmar. PLoS ONE.

[5] Zhou P., Yang X.-L., Wang X.-G., Hu B., Zhang L., Zhang W., Si H.-R., Zhu Y., Li B., Huang C.-L. (2020). A pneumonia outbreak associated with a new coronavirus of probable bat origin. Nature.

[6] Mollentze N., Streicker D.G. (2020). Viral zoonotic risk is homogenous among taxonomic orders of mammalian and avian reservoir hosts. Proc. Natl. Acad. Sci. USA.

[7] Skirmuntt E.C., Escalera-Zamudio M., Teeling E.C., Smith A., Katzourakis A. (2020). The Potential Role of Endogenous Viral Elements in the Evolution of Bats as Reservoirs for Zoonotic Viruses. Annu. Rev. Virol..

[8] Pauwels R. (1986). The clinical relevance of airway inflammation. Eur. J. Respir. Dis. Suppl..

[9] Woda A., Picard P., Dutheil F. (2016). Dysfunctional stress responses in chronic pain. Psychoneuroendocrinology.

[10] Ahn M., Anderson D.E., Zhang Q., Tan C.W., Lim B.L., Luko K., Wen M., Chia wn Mani S., Wang L.C., Ng J.H.J. (2019). Dampened NLRP3-mediated inflammation in bats and implications for a special viral reservoir host. Nat. Microbiol..

[11] Mann D.L. (2020). SARS-CoV-2 and Bats: From Flight to Fighting COVID-19. JACC Basic Transl. Sci..

[12] Weimerskirch H., Guionnet T., Martin J., Shaffer S.A., Costa D.P. (2000). Fast and fuel efficient? Optimal use of wind by flying albatrosses. Proc. R. Soc. B Biol. Sci..

[13] Schmidt-Rohr K. (2020). Oxygen Is the High-Energy Molecule Powering Complex Multicellular Life: Fundamental Corrections to Traditional Bioenergetics. ACS Omega.

[14] Korla K. (2016). Reactive oxygen species and energy machinery: An integrated dynamic model. J. Biomol. Struct. Dyn..

[15] Banerjee A., Baker M.L., Kulcsar K., Misra V., Plowright R., Mossman K. (2020). Novel Insights Into Immune Systems of Bats. Front. Immunol..

[16] 13 Awesome Facts About Bats 2016. https://www.doi.gov/blog/13-facts-about-bats.

[17] (2020). The Lives of Bats. https://www.biologicaldiversity.org/campaigns/bat_crisis_white-nose_syndrome/the_lives_of_bats.html#:~:text=Bats%20are%20the%20only%20true,bat%2C%20which%20feeds%20on%20blood.

[18] De Sabato L., Lelli D., Faccin F., Canziani S., Di Bartolo I., Vaccari G., Moreno A. (2019). Full genome characterization of two novel Alpha-coronavirus species from Italian bats. Virus Res..

[19] Li W., Shi Z., Yu M., Ren W., Smith C., Epstein J.H., Wang H., Crameri G., Hu Z., Zhang H. (2005). Bats are natural reservoirs of SARS-like coronaviruses. Science.

[20] Moyo S., Jacobs D.S. (2020). Faecal analyses and alimentary tracers reveal the foraging ecology of two sympatric bats. PLoS ONE.

[21] Vesterinen E., Puisto A.I.E., Blomberg A.S., Lilley T.M. (2018). Table for five, please: Dietary partitioning in boreal bats. Ecol. Evol..

[22] Castro L.S., Dorval M.E., Matheus L.M., Bednaski A.V., Facco G.G., Silveira M., Santos C.F., Gontijo C.M., Oliveira A.P.G., Ferreira E.C. (2020). Leishmania presence in bats in areas endemic for leishmaniasis in central-west Brazil. Int. J. Parasitol. Parasites Wildl..

[23] Götze S., Denzinger A., Schnitzler H.-U. (2020). High frequency social calls indicate food source defense in foraging Common pipistrelle bats. Sci. Rep..

[24] Liang J., Yang J., Xie H., Peng X., He X., Sun Y., Zhang L. (2019). Impact of external odor on self-grooming of lesser flat-headed bats, Tylonycteris pachypus. Ecol. Evol..

[25] Seim I., Fang X., Xiong Z., Lobanov A.V., Huang Z., Ma S., Feng Y., Turanov A.A., Zhu Y., Lenz T.L. (2013). Genome analysis reveals insights into physiology and longevity of the Brandt’s bat Myotis brandtii. Nat. Commun..

[26] Whittemore K., Martínez-Nevado E., Blasco M.A. (2019). Slower rates of accumulation of DNA damage in leukocytes correlate with longer lifespans across several species of birds and mammals. Aging.

[27] Venn-Watson S., Jensen E.D., Smith C.R., Xitco M., Ridgway S. (2015). Evaluation of annual survival and mortality rates and longevity of bottlenose dolphins (Tursiops truncatus) at the United States Navy Marine Mammal Program from 2004 through 2013. J. Am. Vet. Med. Assoc..

[28] Widagdo W., Sooksawasdi Na Ayudhya S., Hundie G.B., Haagmans B.L. (2019). Host determinants of MERS-CoV transmission and pathogenesis. Viruses.

[29] Caron A., Bourgarel M., Cappelle J., Liegeois F., De De Nys H., Roger F. (2018). Ebola Virus Maintenance: If Not (Only) Bats, What Else?. Viruses.

[30] Fenton M.B., Streicker D.G., Racey P.A., Tuttle M.D., Medellin R.A., Daley M.J., Recuenco S., Bakker K.M. (2020). Knowledge gaps about rabies transmission from vampire bats to humans. Nat. Ecol. Evol..

[31] Zhao H. (2020). COVID-19 drives new threat to bats in China. Science.

